# Metagenomic Analysis of Bacteria, Fungi, Bacteriophages, and Helminths in the Gut of Giant Pandas

**DOI:** 10.3389/fmicb.2018.01717

**Published:** 2018-07-31

**Authors:** Shengzhi Yang, Xin Gao, Jianghong Meng, Anyun Zhang, Yingmin Zhou, Mei Long, Bei Li, Wenwen Deng, Lei Jin, Siyue Zhao, Daifu Wu, Yongguo He, Caiwu Li, Shuliang Liu, Yan Huang, Hemin Zhang, Likou Zou

**Affiliations:** ^1^Department of Applied Microbiology, College of Resources, Sichuan Agricultural University, Chengdu, China; ^2^Department of Nutrition and Food Science, University of Maryland, College Park, College Park, MD, United States; ^3^College of Life Sciences, Sichuan University, Chengdu, China; ^4^The China Conservation and Research Center for the Giant Panda, Wolong, China; ^5^College of Food Science, Sichuan Agricultural University, Ya’an, China; ^6^Key Laboratory of State Forestry and Grassland Administration on Conservation Biology of Rare Animals in The Giant Panda National Park (China Conservation and Research Center of Giant Panda), Wolong, China

**Keywords:** bacteria, fungi, bacteriophages, helminths, giant pandas, metagenomic sequencing

## Abstract

To obtain full details of gut microbiota, including bacteria, fungi, bacteriophages, and helminths, in giant pandas (GPs), we created a comprehensive microbial genome database and used metagenomic sequences to align against the database. We delineated a detailed and different gut microbiota structures of GPs. A total of 680 species of bacteria, 198 fungi, 185 bacteriophages, and 45 helminths were found. Compared with 16S rRNA sequencing, the dominant bacterium phyla not only included Proteobacteria, Firmicutes, Bacteroidetes, and Actinobacteria but also Cyanobacteria and other eight phyla. Aside from Ascomycota, Basidiomycota, and Glomeromycota, Mucoromycota, and Microsporidia were the dominant fungi phyla. The bacteriophages were predominantly dsDNA Myoviridae, Siphoviridae, Podoviridae, ssDNA Inoviridae, and Microviridae. For helminths, phylum Nematoda was the dominant. In addition to previously described parasites, another 44 species of helminths were found in GPs. Also, differences in abundance of microbiota were found between the captive, semiwild, and wild GPs. A total of 1,739 genes encoding cellulase, β-glucosidase, and cellulose β-1,4-cellobiosidase were responsible for the metabolism of cellulose, and 128,707 putative glycoside hydrolase genes were found in bacteria/fungi. Taken together, the results indicated not only bacteria but also fungi, bacteriophages, and helminths were diverse in gut of giant pandas, which provided basis for the further identification of role of gut microbiota. Besides, metagenomics revealed that the bacteria/fungi in gut of GPs harbor the ability of cellulose and hemicellulose degradation.

## Introduction

Mammals are metagenomic because they consist of their own gene complements and a large number of microorganisms residing within them (Ley et al., 2008). These microorganisms include bacteria, fungi, protozoa, and viruses and are collectively referred to as microbiota (Kamada et al., 2013). The gut microbiota is an extra organ that regulates the immune system and influences the physiology, health, and nutrition of its host (Maurice et al., 2013; Qin et al., 2014; Zhernakova et al., 2016). It not only makes an essential contribution to physiology and metabolism of host (Bäckhed et al., 2004; Hooper and Macpherson, 2010; Tremaroli and Backhed, 2012) but also serves as an environmental factor that contributes to obesity and its comorbidities (Tremaroli and Backhed, 2012) and protects the gut against invasion of exogenous pathogens (Kamada et al., 2013).

Compared with other members of microbiota, bacteria are abundant and therefore more influential on the gut of mammals (Human Microbiome, and Project, 2012), particularly related to health and disease of host (Kelly et al., 2005). Fungi also play an important role in disease of host (Huffnagle and Noverr, 2013; Sokol et al., 2017) and may be responsible for fiber degradation and fermentative digestion in herbivores (Li and Heath, 1993; Liggenstoffer et al., 2010). Bacteriophages are the most abundant biological group on earth and genetically even more diverse than their bacterial prey/hosts (Reyes et al., 2013). They are the most abundant members of the gut virobiota (Łusiak-Szelachowska et al., 2017) contributing to gut inflammation and bacterial dysbiosis (Norman et al., 2015). Parasitic helminths (PHs) residing in the gut have profound potentials to threaten the stability and persistence of hosts, impair host fitness, and even lead to mortality of hosts. The interactions between gut PHs and commensal bacteria are likely to play a pivotal role in regulating the development of the gut immune system (Cantacessi et al., 2014; Lee et al., 2014).

The giant panda (GP, *Ailuropoda melanoleuca*) is a rare endemic wild animal found in China and a flagship species for wildlife conservation. GP belongs to the order Carnivora with a typical carnivorous digestive system (Fang et al., 2012; Xue et al., 2015), but almost entirely lives on a bamboo-dominated diet. Its genome encodes all the enzymes necessary for a carnivorous digestive system but lacks those for digesting cellulose and hemicellulose (Fang et al., 2012). However, low digestibility of cellulose and hemicellulose for the GP’s unique bamboo diet may be assisted by microbiota (Zhu et al., 2011; Xue et al., 2015). Necessary enzymes for digestion of cellulose include cellulase (EC 3.2.1.4), cellulose 1,4-β-cellobiosidase (EC 3.2.1.91), and β-glucosidases (EC 3.2.1.21) (Kubicek et al., 2009; Zhu et al., 2011).

To date, most studies have been focusing on diversity of gut bacteria of GPs, and thus little is known about the characteristics of the fungi, bacteriophages, and helminths. The present study was to provide full details of gut microbiota of the GPs, including bacteria, fungi, bacteriophages and helminths, established by metagenomic sequencing. We also provided a detailed characterization of the bacteria and fungi possessing the genes associated with cellulose degradation and glycoside hydrolase (GH) to evaluate the ability of cellulose, hemicellulose and starch hydrolysis of microbiota.

## Materials and Methods

### Sample Collection

Fecal samples from the captive, semiwild, and wild GPs were collected immediately after defecation, snap frozen, and shipped to the laboratory on dry ice. Four samples from the captive GPs collected from the China Conservation and Research Center for the Giant Panda, six samples of semiwild GPs from Hetaoping Base and Liziping Nature Reserve and three samples from the wild GPs from Wolong Nature Reserve were included. Captive, semiwild, and wild GPs live in different environments. Captive GPs lived in man-made limited space for attracting tourists during visits, whereas semiwild GPs lived in a large area and natural habitat or reintroduced into primeval forest without any disturbance by humans. Wild GPs were born and lived in the wild.

### Extraction of DNA and Sequencing

DNA was extracted from fecal samples by using the PowerFecal^®^ DNA Isolation Kit (MO BIO Laboratories, Inc.) following the manufacturer’s instructions. The metagenomic sequencing was performed on an Illumina NextSeq 500 platform in a 2 × 150 paired-end mode.

### Annotation of Microbial Genome

An in-house comprehensive microbial genome database (CMDB) was created using scaffold-level or chromosome-level genomic data from National Center for Biotechnology Information (NCBI) GenBank/Refseq, which spans 16,574 species including 6,761 bacteria, 1,641 fungi, 456 protozoa, 7,149 viruses, and 567 archaea. The high-quality short reads were aligned against the CMDB. Genomes of PH, including 94 from parasitic nematodes and 29 from parasitic trematodes, were downloaded from wormbase parasite ftp site^1^ (Howe et al., 2016) (WBPS8). The WBPS8 also includes eight free-living *Caenorhabditis* worms. Additional genomes, including genomes of human, GP, bamboo, and flies, were included in the analysis for contamination screening and comparison.

Whole metagenome shotgun sequencing reads from GP fecal samples were first subject to quality processing by using the in-house scripts to trim the adaptors, low quality, and duplication reads. Reads with low complexity or length < 90 bp were then removed. *In silico* decontamination was performed by mapping those processed reads to the potential contamination genomes. The final remaining cleaned reads were used for microbial profiling. If the bacteria, bacteriophages, fungi, and PH genome were uniquely mapped by the >50, 50, 300, and 1800 cleaned reads from a sample, the corresponding bacteria, bacteriophages, fungi, and PH species were considered to be present in this sample. The different cutoffs were selected for fungi and PH because their genomes are generally larger compared with the bacterium/bacteriophage. Abundance of a given genome within a sample was obtained by adding all the mapped reads specific to this genome from the corresponding sample.

### Prediction of Functional Genes

These non-redundant reads were performed *de novo* assembly by using Soapdenovo 2 (Luo et al., 2012) to obtain long contigs and scaffolds. The predicted open reading frames were annotated and compared with BLASTP databases by using MetaGeneMark (Zhu et al., 2010) from the long contigs with a length more than 300 bp. These non-redundant protein sequences were compared in NCBI-NR database with 90% similarity using CD-HIT software (Li and Godzik, 2006). The genes were functionally annotated using the Kyoto Encyclopedia of Genes and Genomes (KEGG) database (Kanehisa et al., 2017) and Carbohydrate-Active Enzymes (CAZy) database (Cantarel et al., 2009).

### Analysis of Data

The cladogram with circular representations of taxonomic and phylogenetic trees was produced using GraPhlAn (Asnicar et al., 2015). The network displayed correlations between different species by using Cytoscape (Shannon et al., 2003). KEGG metabolic pathways were generated to obtain the most comprehensive image using ipath 2.0 (Yamada et al., 2011).

## Results

### Metagenomic Sequencing of DNA

The 124.6 gigabases (Gbs) of high-quality reads was obtained from all samples, with an average of 9.6 Gbs of each sample (Supplementary Table S1). An average of 10.3 Gbs came from the captive GPs, 7.7 Gbs from semiwild GPs, and 12.5 Gbs from wild GPs. The short reads for each individual sample were performed *de novo* assembly into long contigs for further analysis and annotation. Totally, 2.3 million contigs and a total sequence length of 1,199 million bps were obtained eventually with an average of 602 contig N50, 7.0% of which (159.1 thousand sequences) were more than 1,000 bp (Supplementary Table S2).

We performed alignment of all high-quantity reads against CMDB and WBPS8 to profile diversity of microbiota in the gut of GPs. After exclusion of the irrelevant reads with bacteria, fungi, bacteriophages, and helminths, 316.2 million reads were obtained. Among them, 231.7 million reads (73.28%) belonged to bacterial genomes, 5.6 million reads (1.77%) to fungal genomes, 3.7 million reads (1.17%) to bacteriophage genomes, and 75.2 million reads (23.78%) to helminthous genomes.

### Diversity of Bacteria

**FIGURE 1:**
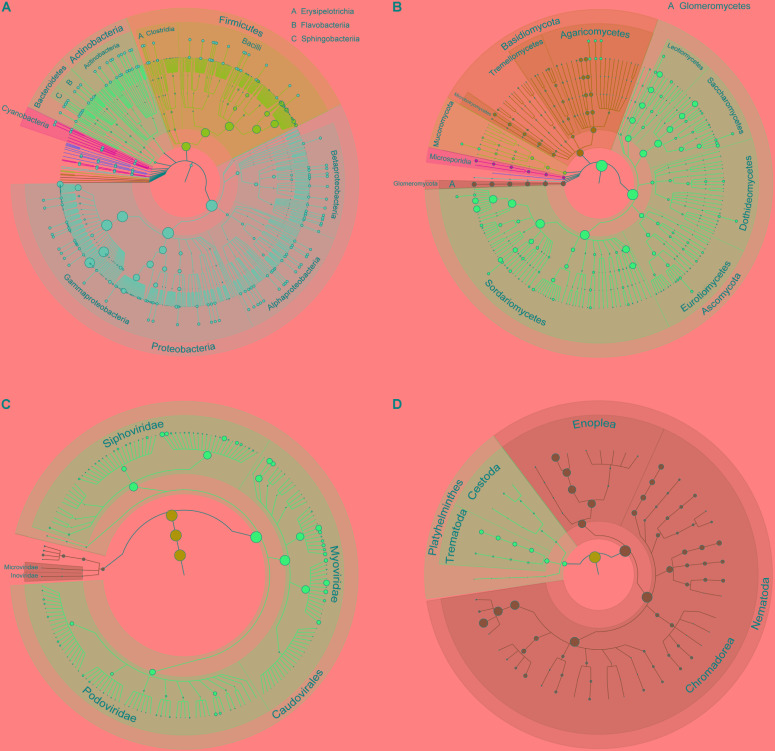
Taxonomic tree of bacteria, fungi, bacteriophages, and helminths in the gut of GPs. **(A)** Bacteria; **(B)** fungi; **(C)** bacteriophages; and **(D)** helminths. From the inner to outer circles, the taxonomic levels range from kingdom to species. The diameter of nodes indicates the abundance at different taxonomic levels, and different colors denote different taxonomic clades.

**FIGURE 2:**
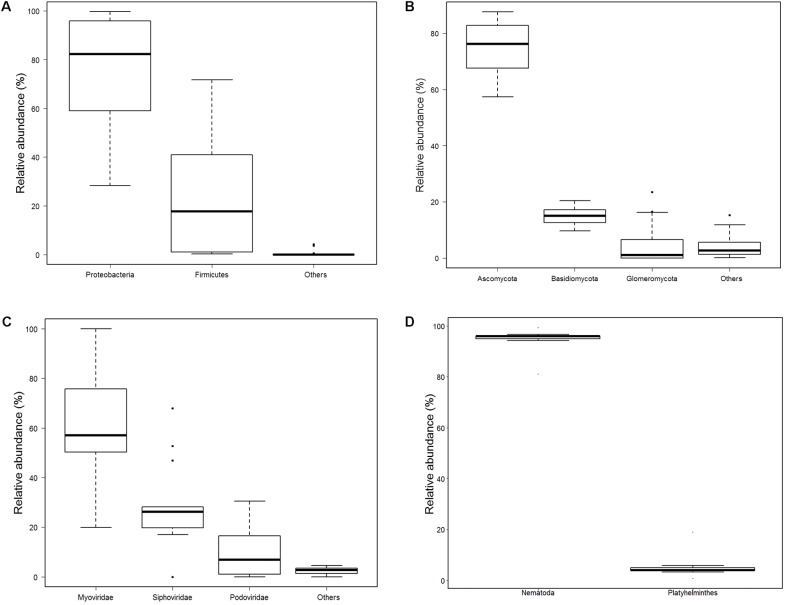
Relative abundance of bacteria, fungi, bacteriophages, and helminths at phyla level. **(A)** Bacteria; others included *Bacteroidetes*, *Actinobacteria*, *Cyanobacteria*, Tenericutes, Candidatus Saccharibacteria, Acidobacteria, Spirochaetes, Deinococcus Thermus, Verrucomicrobia, Fusobacteria, Gemmatimonadetes, and one unclassified phylum. **(B)** Fungi; others included Mucoromycota, Microsporidia, Chytridiomycota, and Zoopagomycota. **(C)** Bacteriophages; Others included *Microviridae*, *Inoviridae*, and 2 unclassified phyla. **(D)** helminths.

**FIGURE 3:**
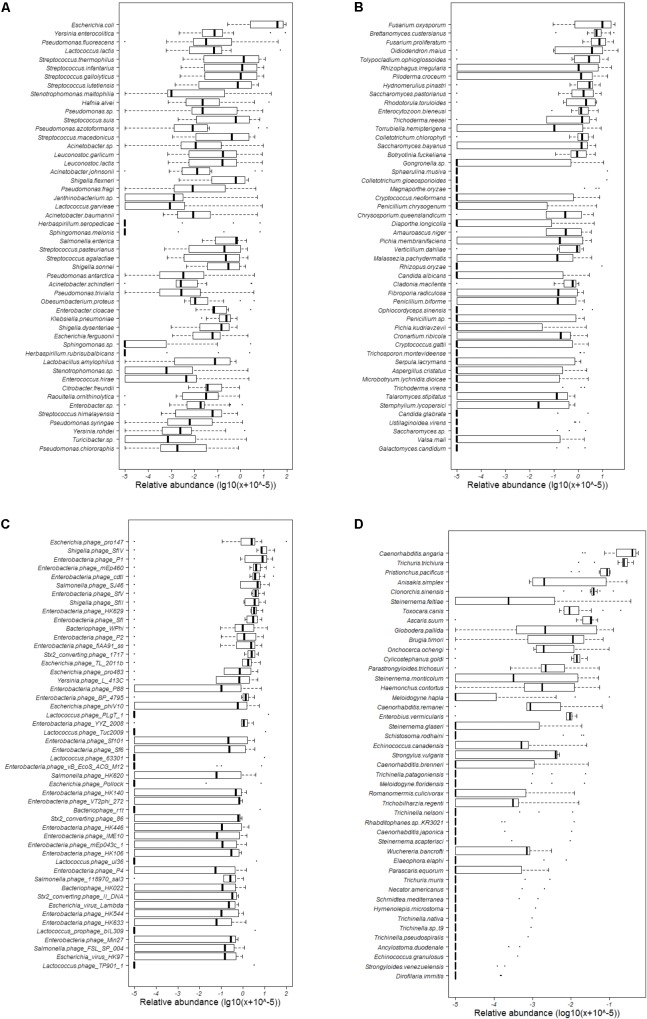
Abundance of the 50 most abundant bacterial, fungal, and bacteriophagic species and all helminthous species. **(A)** Bacteria; **(B)** fungi; **(C)** bacteriophages; and **(D)** helminths.

Totally, 680 species of bacteria affiliated with 13 classified phyla, 23 classified classes, 47 classified orders, 88 classified families, 228 classified genera, 1 unclassified phylum, 4 unclassified classes, 5 unclassified orders, 9 unclassified families, and 10 unclassified genera that could not be classified to the corresponding taxonomic level were found (**Figure 1A**). The two most abundant phyla were Proteobacteria (75.41%) and Firmicutes (23.94%), followed by *Bacteroidetes* (0.52%), Actinobacteria (0.09%), and Cyanobacteria (0.02%), and others (0.02%) (Supplementary Table S3 and **Figure 2A**). Within the Proteobacteria, three top abundant classes were the members of Gammaproteobacteria with an average abundance of 72.9%, Betaproteobacteria with 1.7%, and Alphaproteobacteria with 0.8%. Within the Firmicutes, the most abundant class was Bacilli with an average of 23.3%, followed by Clostridia (0.5%) and Erysipelotrichia (0.1%). The phylum Bacteroidetes primarily consisted of Sphingobacteriia (0.27%) and Flavobacteriia (0.25%). Actinobacteria (0.1%) was the only class in the phylum Actinobacteria. Two classes, namely, Gloeobacteria (0.0029%) and Hormogoneae (0.00009%), mainly constituted the phylum Cyanobacteria. At the genus level, 50 most abundant genera are shown in Supplementary Figure S1. The top 10 prevalent genera were *Escherichia* (41.1%), *Streptococcus* (15.6%), *Pseudomonas* (10.7%), *Yersinia* (8.9%), *Lactococcus* (4.8%), *Acinetobacter* (3.5%), *Leuconostoc* (2.1%), *Stenotrophomonas* (2.0%), *Hafnia* (1.7%), and *Shigella* (1.6%) (Supplementary Table S4). The 50 most abundant species are shown in **Figure 3A**. The top 10 abundant species were *Escherichia coli* (40.8%), *Yersinia enterocolitica* (8.3%), *Pseudomonas fluorescens* (4.9%), *Lactococcus lactis* (4.2%), *Streptococcus thermophilus* (3.5%), *Streptococcus infantarius* (2.9%), *Streptococcus gallolyticus* (2.8%), *Streptococcus lutetiensis* (1.8%), *Stenotrophomonas maltophilia* (1.7%), and *Hafnia alvei* (1.7%) (Supplementary Table S5).

We also analyzed the correlation of 58 genera with an average abundance of ≥0.01% based on Pearson coefficient of correlation (Supplementary Figure S2). Obviously, these genera were affiliated with the phyla Proteobacteria, Firmicutes, Bacteroidetes, Actinobacteria, and Cyanobacteria. Positive correlations were observed between the majority of genera. The genus *Ralstonia* showed negative correlations with *Escherichia*, *Shigella*, *Salmonella*, *Klebsiella*, and an unclassified genus of *Enterobacteriaceae.* The genus *Bacillus* had a negative correlation with *Serratia* and *Carnobacterium.*

To evaluate the bacterial diversity in the gut of GPs from different environments, we analyzed the average abundance of bacteria at the phylum level (Supplementary Figure S3). The results showed no significant differences in the abundance of Proteobacteria and Firmicutes between GPs from different environments. However, the abundance of both Bacteroidetes and Actinobacteria in the gut of wild GPs were significantly higher than that in the gut of the captive and semiwild GPs (*p* < 0.05). The phylum Cyanobacteria was only detected in semiwild. Besides, we evaluated the difference at genus level with more than 0.01% among the captive, semiwild, and wild GPs (Supplementary Figure S4). Thirty genera showed high abundance in the wild GPs, and *Pseudomonas*, *Janthinobacterium*, and *Sphingobacterium* were found significantly high (*p* < 0.05). The abundances of 15 genera were high in the captive GPs, and *Raoultella* was significantly high (*p* < 0.05). However, other genera showed high abundance in the semiwild GPs.

### Diversity of Fungi

In total, 198 species of fungi from 7 classified phyla, 17 classified classes, 44 classified orders, 87 classified families, 130 classified genera, 3 unclassified classes, 3 unclassified orders, and 4 unclassified families were detected (**Figure 1B**). The most abundant phylum was Ascomycota (75.5%), followed by Basidiomycota (14.7%), Glomeromycota (5.4%), Mucoromycota (2.3%), Microsporidia (2.0%), and others (0.1%) (**Figure 2B**). Within Ascomycota, five most abundant classes, namely, Sordariomycetes (40.0%), Saccharomycetes (16.7%), Leotiomycetes (10.7%), Eurotiomycetes (5.2%), and Dothideomycetes (2.3%), of more than 1% were observed. Three classes, namely, Agaricomycetes (8.1%), Microbotryomycetes (2.8%), and Tremellomycetes (2.0%), belonged to the phylum Basidiomycota. The Glomeromycetes (5.4%) dominated in Glomeromycota. An unclassified class (2.3%) belonged to Mucoromycota, and an unclassified class (2.0%) belonging to Microsporidia were also found.

At the genus level, 50 most abundant genera are shown in Supplementary Figure S5. The *Fusarium* (22.6%), *Brettanomyces* (9.6%), *Oidiodendron* (9.1%), *Tolypocladium* (5.5%), *Rhizophagus* (5.4%), *Saccharomyces* (4.6%), *Piloderma* (3.4%), *Colletotrichum* (3.2%), *Hydnomerulius* (3.0%), and *Rhodotorula* (2.5%) were the top 10 genera (Supplementary Table S4). In addition, 50 most abundant species are shown in **Figure 3B**. The top 10 species were *Fusarium oxysporum* (17.5%), *Fusarium proliferatum* (11.2%), *Brettanomyces custersianus* (11.0%), *Oidiodendron maius* (9.3%), *Rhizophagus irregularis* (6.6%), *Tolypocladium ophioglossoides* (6.0%), *Piloderma croceum* (3.5%), *Hydnomerulius pinastri* (3.4%), *Rhodotorula toruloides* (3.3%), and *Saccharomyces pastorianus* (3.2%) (Supplementary Table S5).

We also analyzed the correlation of 110 genera with an average abundance ≥0.01% based on Pearson coefficient of correlation (Supplementary Figure S6). Obviously, positive correlations were observed between the majorities of genera. However, negative correlations between several special genera were also found. The *Hydnomerulius* showed negative correlations with 12 genera. The *Rhodotorula* showed negative correlations with *Hydnomerulius*, *Oidiodendron*, *Piloderma*, and *Tolypocladium*.

We also analyzed the correlation of 58 bacteria genera with an average abundance of ≥0.01% and 110 fungi genera with an average abundance ≥0.01% based on Pearson coefficient of correlation (Supplementary Figure S7). Obviously, positive correlations were observed between the majorities of genera. However, several fungi genera, including *Brettanomyces*, *Cladonia*, *Fusarium*, *Hydnomerulius*, *Rhodotorula*, *Tolypocladium*, and *Verticillium*, had negative correlation with many bacteria. Several bacteria genera, including *Streptococcus*, *Pectobacterium*, *Lactobacillus*, *Klebsiella*, *Eubacterium*, *Escherichia*, *Carnobacterium* and *Anaerostipes*, had negative correlation with many fungi.

We found no significant differences between the three groups from different environments at the phylum level (*p* > 0.05) (Supplementary Figure S8). Subsequently, we analyzed the 57 fungus genera with an average abundance of more than 0.01% among captive, semiwild, and wild GPs (Supplementary Figure S9). The abundance of 18 genera were high in the captive GPs. Eighteen genera were high in the wild GPs, and *Gongronella* and *Ophiocordyceps* were significantly high (*p* < 0.05). Other genera were high in the semiwild GPs, and *Rhodotorula* was also significantly high (*p* < 0.05).

### Diversity of Bacteriophages

A total of 185 bacteriophages from 1 classified and 1 unclassified orders, 5 classified and 2 unclassified families, and 23 classified and 6 unclassified genera were obtained (**Figure 1C**). Most of bacteriophages (97.9%) came from the order Caudovirales, whereas others came from unclassified order (2.1%). Aside from an unclassified families accounting for 2.1%, the five classified families were Myoviridae (58.9%), Siphoviridae (28.8%), Podoviridae (10.0%), Inoviridae (0.1%), and Microviridae (0.07%) (**Figure 2C**).

The relative abundance of all 29 genera is shown in Supplementary Figure S10. The top 10 abundant genera were Lambdavirus (22.7%), P2 virus (21.9%), P1 virus (13.8%), Epsilon15 virus (3.3%), P22 virus (1.9%), Nona33 virus (0.7%), Rtp virus (0.6%), G7c virus (0.3%), Tl2011 virus (0.3%), and T4 virus (0.2%), (Supplementary Table S4). Twenty-two species of bacteriophages showed more than 1% abundance (**Figure 3C**), and 10 most abundant species were *Escherichia* phage pro147 (10.1%), *Shigella* phage SfIV (9.9%), *Enterobacteria* phage P1 (8.9%), *Enterobacteria* phage mEp460 (6.2%), *Enterobacteria* phage cdtI (5.8%), *Salmonella* phage SJ46 (5.0%), *Enterobacteria* phage SfV (3.9%), *Shigella* phage SfII (3.8%), *Enterobacteria* phage HK629 (3.5%), and *Enterobacteria* phage SfI (3.2%) (Supplementary Table S5). Analyzing the host of bacteriophage showed that most of bacteriophages were from *Escherichia* (35.7%), followed by *Enterobacteria* (20.5%), *Salmonella* (17.8%), *Shigella* (7.0%), and *Lactococcus* (4.3%). Two prophages, namely, *Lactococcus prophage* bIL286 and *Lactococcus prophage* bIL309, were also found.

We analyzed the correlations of 26 genera based on Pearson coefficient of correlation (Supplementary Figure S11). Positive correlations were observed among all genera. Meanwhile, the family Microviridae was only detected in semiwild GPs, and no significant difference in the abundance of Myoviridae, Siphoviridae, Podoviridae, and Inoviridae among the captive, semiwild, and wild GPs was observed (*p* > 0.05) (Supplementary Figure S12). Interestingly, the analysis phage genera showed a difference among captive, semiwild, and wild GPs (Supplementary Figure S13). Most genera were more abundant in the gut of captive GP than in semiwild and wild GPs.

### Diversity of Helminths

A total of 45 helminths were identified from 2 classified phyla, 4 classified classes, 1 unclassified class, 12 classified orders, 23 classified families, and 32 classified genera (**Figure 1D**). The phylum Nematoda was dominant (94.8%), and *Platyhelminthes* only accounted for 5.2% (**Figure 2D** and Supplementary Table S3). The phylum Nematoda contained the class Chromadorea with abundance of 70.6% and Enoplea with 24.2%. The phylum Platyhelminthes had three classes, namely, Trematoda (4.8%), Cestoda (0.3%), and 1 unclassified class (0.01%). At the genus level, all 32 abundant genera are shown in Supplementary Figure S14. The 10 most abundant genera were *Caenorhabditis* (35.2%), *Trichuris* (23.7%), *Pristionchus* (7.5%), *Anisakis* (5.9%), *Steinernema* (4.8%), *Clonorchis* (4.3%), *Toxocara* (3.0%), *Ascaris* (2.8%), *Globodera* (2.3%), and *Brugia* (1.6%) (Supplementary Table S4).

**FIGURE 4:**
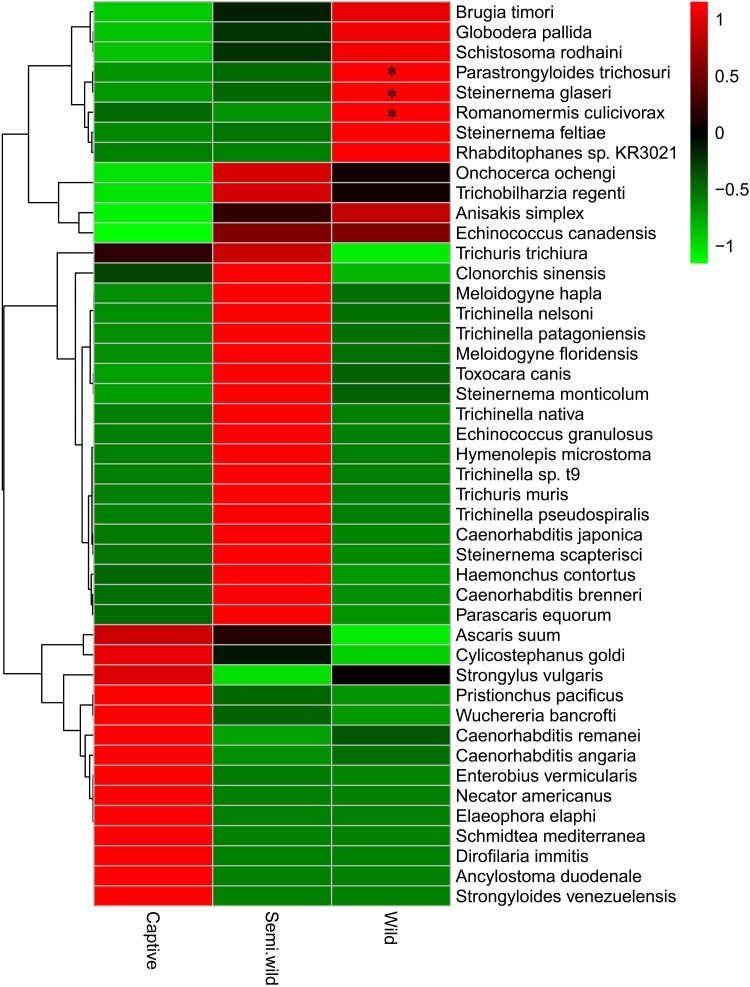
Average abundance of all helminths between three different groups. ^∗^Significant difference (*p* < 0.05).

At the species level, the most dominant helminth was *Caenorhabditis angaria* (34.0%), followed by *Trichuris trichiura* (23.7%), *Pristionchus pacificus* (7.5%), *Anisakis simplex* (5.9%), *Clonorchis sinensis* (4.3%), *Steinernema feltiae* (3.4%), *Toxocara canis* (3.0%), *Ascaris suum* (2.8%), *Globodera pallida* (2.3%), and *Brugia timori* (1.6%) (**Figure 3D**). In all 45 helminths, 30 PH were found in mammals or humans (**Figure 4** and **Table 1**).

**Table 1:** Species of helminths in the gut of GPs in this study.

Family	Parasitic helminths	Potential host	Reference
Alloionematidae	*Rhabditophanes* sp. KR3021	Free-living	Willems et al., 2005
Ancylostomatidae	*Ancylostoma duodenale*	Humans	Chowdhury and Schad, 1972
	*Necator americanus*	Humans	Chow et al., 2000
Anisakidae	*Anisakis simplex*	Humans	Baeza et al., 2004
Ascarididae	*Ascaris suum*	Pigs	Liu et al., 2012
	*Parascaris equorum*	Horses	Lyons and Tolliver, 2004
Dugesiidae	*Schmidtea mediterranea*	Free-living	Egger et al., 2007
Haemonchidae	*Haemonchus contortus*	Ruminants	Peter and Chandrawathani, 2005
Heteroderidae	*Globodera pallida*	Plants	Williamson and Hussey, 1996
Hymenolepididae	*Hymenolepis microstoma*	Rodents	Macnish et al., 2003
Meloidogynidae	*Meloidogyne floridensis*	Plants	Church, 2007
	*Meloidogyne hapla*	Plants	Opperman et al., 2008
Mermithidae	*Romanomermis culicivorax*	Insects	Powers et al., 1986
Neodiplogasteridae	*Pristionchus pacificus*	Free-living	Gutierrez and Sommer, 2004
Onchocercidae	*Brugia timori*	Humans	McReynolds et al., 1986
	*Dirofilaria immitis*	Dogs	Kramer et al., 2005
	*Elaeophora elaphi*	Red deer	Hofle et al., 2004
	*Onchocerca ochengi*	Cattles	Trees, 1992
	*Wuchereria bancrofti*	Humans	Gnanasekar et al., 2002
Opisthorchiidae	*Clonorchis sinensis*	Humans	Wang et al., 2011
Oxyuridae	*Enterobius vermicularis*	Humans	Cook, 1994
Rhabditidae	*Caenorhabditis angaria*	Free-living	Gutierrez and Sommer, 2004
	*Caenorhabditis brenneri*	Free-living	Diaz et al., 2010
	*Caenorhabditis japonica*	Free-living	
	*Caenorhabditis remanei*	Free-living	
Schistosomatidae	*Schistosoma rodhaini*	Rodents	Steinauer et al., 2008
	*Trichobilharzia regenti*	Mammals and birds	Lichtenbergova et al., 2011
Steinernematidae	*Steinernema feltiae*	Insects	Popiel et al., 1989
	*Steinernema glaseri*	Insects	Koppenhofer and Kaya, 1995
	*Steinernema monticolum*	Insects	Liu et al., 1997
	*Steinernema scapterisci*	Insects	Grewal et al., 1993
Strongylidae	*Cylicostephanus goldi*	Mammals	Grant et al., 2006
	*Strongylus vulgaris*	Horses	Nielsen et al., 2008
Strongyloididae	*Parastrongyloides trichosuri*	Mammals	Grant et al., 2006
	*Strongyloides venezuelensis*	Mammals and GPs	Sato and Toma, 1990; Zhang et al., 2011
Taeniidae	*Echinococcus canadensis*	Humans	Schneider et al., 2010
	*Echinococcus granulosus*	Humans	Torgerson and Heath, 2003
Toxocaridae	*Toxocara canis*	Mammals and humans	Crompton and Savioli, 1993
Trichinellidae	*Trichinella nativa*	Mammals	Pozio et al., 2001
	*Trichinella nelsoni*	Carnivores	Pozio et al., 1997
	*Trichinella patagoniensis*	Mammals and birds	Krivokapich et al., 2012
	*Trichinella pseudospiralis*	Mammals and birds	
	*Trichinella* sp. t9	Mammals	
Trichuridae	*Trichuris muris*	Mammalians	Hayes et al., 2010
	*Trichuris trichiura*	Humans	Crompton and Savioli, 1993


No significant differences were observed among the captive, semiwild, and wild GPs at the phylum level (*p* > 0.05) (Supplementary Figure S15). As shown in **Figure 4**, 14 species including *C. angaria* and *P. pacificus* were found high in captive GPs. Twenty-two species were found high in semiwild GPs. In addition, nine species were found high in wild GPs. Among which, *Parastrongyloides trichosuri*, *Steinernema glaseri*, and *Romanomermis culicivorax* were significantly high (*p* < 0.05).

### Cellulose Degradation-Related Microbes and Metabolic Pathways

We compared the genes present in bacterial and fungal metagenome based on KEGG database and CAZy database to assess the ability of hydrolyzing cellulose and encoding the CAZy. By analyzing 1,446,631 genes from 13,075 KO based on KEGG database, we found 1739 genes possessing homologous sequences to the genes encoding cellulase (EC 3.2.1.4) (*n* = 364), β-glucosidase (EC 3.2.1.21) (*n* = 1339), and cellulose 1,4-β-cellobiosidase (EC 3.2.1.91) (*n* = 36). All of which belonged to 223 different bacterial and 36 fungal genera.

The genes encoding cellulase came from 125 bacteria and 24 fungi. The 125 bacteria belonged to 73 genera. The most abundant bacterium was *Bacillus* (*n* = 11), followed by *Thermoanaerobacter* (*n* = 7), *Pseudomonas* (*n* = 6), *Serratia* (*n* = 5), *Paenibacillus* (*n* = 5), *Xanthomonas* (*n* = 5), and *Methylobacterium* (*n* = 5). Among the 24 fungi, 7 members belonged to *Aspergillus*, 3 belonged to *Bipolaris*, 2 belonged to *Verticillium*, and 2 belonged to *Neurospora*.

The genes encoding β-glucosidase came from 243 bacteria and 50 fungi. The 243 bacteria belonged to 142 genera. The most abundant bacteria belonged to *Pseudomonas* (*n* = 21), followed by *Bifidobacterium* (*n* = 10), *Xanthomonas* (*n* = 8), *Paenibacillus* (*n* = 7), and *Serratia* (*n* = 6). The 50 fungi belonged to 20 genera, and the most abundant fungi were from *Bipolaris* (*n* = 3), *Neurospora* (*n* = 2), *Aspergillus* (*n* = 2), and *Fusarium* (*n* = 2).

The genes encoding cellulose 1,4-β-cellobiosidase came from 11 bacteria and 14 fungi. The 11 bacteria belonged to 8 genera, including *Streptomyces* (*n* = 3) and *Cellulomonas* (*n* = 2). The 14 fungi belonged to 12 genera, including *Bipolaris* (*n* = 2) and *Neurospora* (*n* = 2).

In total, we found 128,707 putative GH gene of 117 GH families in all 145 GH families of CAZy database. The 27 GH families were more than 1%, including GH13 (*n* = 12,475), GH23 (*n* = 12,355), GH3 (*n* = 10,253), GH1 (*n* = 6,655), and GH2 (*n* = 6,548) (Supplementary Table S6). A total of 30,209 genes encoding the cellulase, β-glucosidase, and cellulose β-1,4-cellobiosidase were also included (Supplementary Table S7).

**FIGURE 5:**
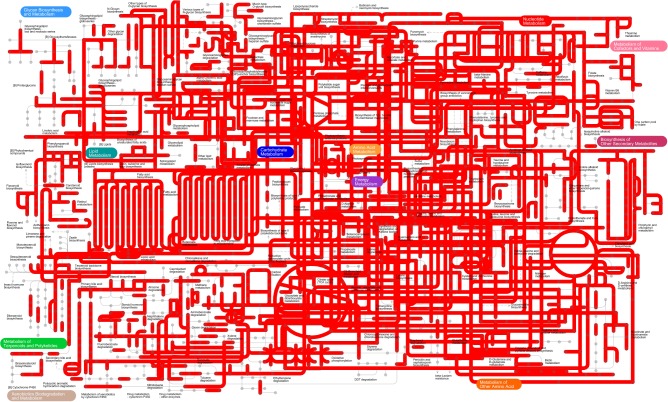
The KEGG pathways determined using iPath of the gut microbiome.

Kyoto Encyclopedia of Genes and Genome metabolic pathways were generated based on metagenomics to obtain the most comprehensive image of gut microbe metabolism in GPs, suggesting that gut microbiome of GPs features enriched activity for metabolism of carbohydrates, nucleotides, lipids, amino acids, energy, terpenoids, polyketides, glycan, cofactors, vitamins and xenobiotics biodegradation, and biosynthesis of other secondary metabolites (**Figure 5**).

## Discussion

At the phylum level, 14 bacterial groups were identified; this finding was more than six and nine phyla found in GPs using 16S rRNA sequencing (Zhu et al., 2011; Xue et al., 2015). As described previously, Proteobacteria and Firmicutes constituted the vast majority of bacteria in the gut of GPs (Zhu et al., 2011; Fang et al., 2012; Tun et al., 2014; Xue et al., 2015). Unlike the published 16S rRNA sequencing data (Zhu et al., 2011; Fang et al., 2012; Tun et al., 2014; Xue et al., 2015), our metagenomic shotgun sequencing study revealed significantly higher abundance of phyla Proteobacteria than that of Firmicutes. Similarly, the two phyla Firmicutes and Proteobacteria were also the predominant phyla in the gut of both brown bear (*Ursus arctos*) (Sommer et al., 2016) and Asiatic black bear (*Ursus thibetanus*) (Song et al., 2017). GP still has a similarity of gut bacteria with other bears in spite of a bamboo diet (Ley et al., 2008). Members of *Bacteroidetes* were also abundant in the gut of GP, as described in cattle (Shanks et al., 2011) and horse (Dougal et al., 2013). In general, the three phyla, namely, Firmicutes, Bacteroidetes, and Proteobacteria, were numerically the most dominant phyla detected in the gut of mammals (Delsuc et al., 2014). Actinobacteria was another phylum that could be found in the gut of humans (Krogius-Kurikka et al., 2009), cheetahs (Becker et al., 2014), and mice (Murphy et al., 2010); Actinobacteria was generally found in adult GPs and absent in geriatric individuals (Tun et al., 2014). The phylum Cyanobacteria also was detected in the gut of GPs (Zhu et al., 2011; Xue et al., 2015). However, it was only detected in semiwild GP in our study. Living environments may influence the abundance of Cyanobacteria in the gut of GPs. *Escherichia* was the most predominant genus in GP’s gut microbiota as described previously. Differently, majority members of abundant genera showed different rankings in the study (Zhu et al., 2011; Xue et al., 2015), e.g., *Streptococcus*, *Pseudomonas*, *Yersinia*, and *Lactococcus* were the most dominant. Besides, bacteria at species level were demonstrated using metagenomic sequencing. As described previously, metagenomic sequencing predicted more microbial species than 16S ribosomal RNA (rRNA) gene sequencing (Zhernakova et al., 2016). Compared with 16S rRNA sequencing technology, we delineated the detailed and different gut bacteria in GPs, especially at species level. As described previously, bacteria belong to phylum Firmicutes possess putative genes coding for cellulose and hemicellulose-digesting enzymes, especially found in species within the *Clostridium* genus (Zhu et al., 2011). It is clear that gut microbial composition data alone cannot resolve whether gut microorganisms of giant pandas are an adaptation to a bamboo diet and aid in the digestion of cellulose and hemicellulose. Fortunately, we identified other potential cellulolytic and hemicellulolytic bacteria in the gut of giant pandas using metagenomics.

The abundance of Bacteroidetes and Actinobacteria in the gut of wild GPs was significantly higher than that in captive and semiwild GPs. Bacteroidetes and Actinobacteria were also discovered as the dominant phyla in the vagina and uterus of GPs (Yang et al., 2017). High-carbohydrate and low-fat diet in natural environments may contribute to the increase in Bacteroidetes (Ley et al., 2006). Bacteroidetes is well-known for the degradation of high-molecular weight organic matter (Thomas et al., 2011; Wu et al., 2011). Besides, the Bacteroidetes are believed to complement eukaryotic genomes with degradation enzymes targeting resistant dietary polymers (Thomas et al., 2011). Members of Actinobacteria were described in *a prior* gut study, and they have also been associated with a variety of environments and conditions (D’Argenio and Salvatore, 2015). Diet and environment may lead the wild GPs to obtain these bacteria (Paul et al., 2016; Snijders et al., 2016).

As described previously, the fungal phyla Ascomycota, Basidiomycota, and Mucoromycota were also dominant in the gut of GPs (Tun et al., 2014). Compared with other mammals, the GPs had a more abundant Glomeromycota and Mucoromycota, which were proved to be an important arbuscular mycorrhizal fungi (Hijri et al., 2007; Spatafora et al., 2016). Besides, the phylum Microsporidia has an important proportion in the gut of GPs. Members of Microsporidia are obligate intracellular eukaryotic parasites (Patrick, 2009). At the genus level, most fungi were obviously related with plants. *Fusarium* (Kistler, 1997; Kolb et al., 2001) and *Colletotrichum* (O’Connell et al., 2012) served as plant pathogens. *Oidiodendron* appeared to exist as saprotrophs and could also form ericoid mycorrhizal associations with Ericaceae hosts (Chambers et al., 2008). *Rhizophagus* is an arbuscular mycorrhizal fungi that form symbiotic relationships with plant (Tisserant et al., 2013). *Piloderma* is a wood ectomycorrhizal fungus (Hagerberg et al., 2005). Besides, the members of *Tolypocladium* were widespread as soil-borne insect pathogens and saprotrophs (Mogensen et al., 2011). This discrepancy can be attributed to dietary differences as GPs may consume different species of bamboo, water, and many other foods in nature, which may cause exposure of GPs to these fungi from the diet and environment. Besides, fungi at species level were demonstrated using metagenomic sequencing. Thus, using metagenomic sequencing, we delineated the detailed gut fungi of GPs.

Still now, little was known about the diversity and role of fungi in the gut of giant pandas. Compared to bacteria, the role of fungi within the intestinal microbiota is poorly understood. Fungi also play an important role in disease of host (Huffnagle and Noverr, 2013; Scott et al., 2013; Sokol et al., 2017) or produce a result of interactions that are relevant to health and gut diseases of host with bacteria (Peleg et al., 2010; Mar Rodriguez et al., 2015). In addition to bacteria, fungi in gut of giant pandas harbor the genes coding for starch, cellulose, and hemicellulose-digesting enzymes and could also aid in the digestion of the substances in bamboo (Zhu et al., 2011).

However, most fungus genera were abundant in semiwild and wild GPs than that in captive GPs. The abundance and difference of these fungi can be attributed to diet consumption. Gut fungi may be responsible for fiber degradation and fermentative digestion in herbivores (Li and Heath, 1993; Liggenstoffer et al., 2010). The gut microbiota of herbivore plays a vital role in hydrolyzing carbohydrate components of the plant cell wall including cellulose and xylan components (Brulc et al., 2009; Hooper and Macpherson, 2010; Hess et al., 2011; Wang et al., 2013). In particular, anaerobic fungi are commonly found in the digestive tract of ruminants and monogastric herbivores (Theodorou et al., 1996; Ho et al., 2000; Ljungdahl, 2008; Haitjema et al., 2014) and capable of producing enzymes that efficiently hydrolyze cellulose and hemicelluloses (Ljungdahl, 2008). However, the crucial anaerobic gut fungi, which affiliate with Class Chytridiomycetes, Order Neocallimastigales, and Family Neocallimastigaceae, commonly harbored in many herbivores to assist hydrolysis of cellulose (Theodorou et al., 1996; Ho et al., 2000; Ljungdahl, 2008; Haitjema et al., 2014), were not found in the gut of GPs in this study.

Majority of bacteriophages was found in the gut of GPs, and most of which belonged to the order Caudovirales in nature (Ackermann, 2003; Lim et al., 2016). Previous study demonstrated an increase in the richness of Caudovirales phages in the gut of patients with inflammatory bowel disease (Norman et al., 2015). Clearly, bacteriophages can influence the behavior and pathogenicity of bacteria, and the interaction effect between bacteriophages and bacteria have a significant impact on host health (Bondy-Denomy and Davidson, 2014). The lysis of bacteria by bacteriophages leads to release of proteins, lipids, and nucleic acids, which could induce gut inflammation (Łusiak-Szelachowska et al., 2017). In our study, host of bacteriophages was largely from Enterobacteriaceae. This phenomenon displayed well the symbiotic or parasitic relationships between the bacteriophages and bacteria. Bacteriophages are known as regulators of the bacterial population in the gut (Brown-Jaque et al., 2016), capable to lyse bacterial cell and may control the bacterial population, influencing bacterial diversity and metabolism (Łusiak-Szelachowska et al., 2017). The bactericidal activity of bacteriophages could be used to treat infections gradually as an alternative or a complement to antibiotic therapy in GPs (Sulakvelidze et al., 2001; Merril et al., 2003; Hermoso et al., 2007). Only two prophages, Lactococcus prophage bIL286 and Lactococcus prophage bIL309, were detected in the gut of GPs. Prophage induction may contribute to the dysbiosis of gut microbiota, changing the ratio of symbionts to pathobionts (Mills et al., 2013). Most genera of bacteriophages were more abundant in the gut of captive GPs than that in the gut of semiwild and wild GP. Living environment is an important factor influencing diversity of phages (Łusiak-Szelachowska et al., 2017).

We demonstrated different gut parasites using metagenomic sequencing compared with previous studies. *Baylisascaris schroederi* is the most common parasitic helminth of GPs (Zhang et al., 2012, 2015; Zhao et al., 2013; Zhou et al., 2013). Meanwhile, *Cryptosporidium* spp. (Wang et al., 2015), *Ancylostoma ailuropodae* (Xie et al., 2017), *Ogmocotyle sikae*, *Toxascaris seleactis*, and *Strongyloides* spp. (Zhang et al., 2011) have also been reported to cause parasitic infection in GPs. However, in addition to previously described parasites in GPs, we first found another 44 helminths in GPs in this study which were not described previously (not including *Strongyloides* spp.). This discrepancy can be attributed to genomes as no data were available in WBPS8 for those parasites found in GPs (*B. schroederi*, *O. sikae*, *A. ailuropodae*, and *T. seleactis*). It is worth noting that the current PH database is derived from 50 Helminth Genome Project^2^ (ref), and prominently biased toward the medically important helminth genomes. Therefore, our results should be more sensible at the family level. Among the 45 helminth whose genomes our reads were mapped to, 30 parasitize mammals or human, strongly suggesting that GPs are important reservoir for parasitic helminths. Several major worldwide helminths, such as *T. trichiura*, *Necator americanus*, *Ancylostoma duodenale*, *Enterobius vermicularis*, and *T. canis* (Bundy and Cooper, 1989; Crompton and Savioli, 1993; Bethony et al., 2006), found in humans also were detected in GPs. In addition, several free-living nematodes, including *C. angaria*, *C. brenneri*, *C. japonica*, *C. remanei*, and *P. pacificus* (Kiontke, 2006; Sommer, 2006; Rödelsperger et al., 2013), were also identified, which were dominant in the gut of GPs. *P. pacificus* was reported as living in close association with beetles (Herrmann et al., 2006; Kiontke, 2006). Actually, the five genomes of beetle were found highly abundant in GP’s gut metagenome sample, which might be derived from the bamboo feeds. Besides, no significant difference was observed for most helminths between the three groups from different environments, suggesting that the parasitic infection was ubiquitous in captive, semiwild, and wild GPs.

The genes encoding cellulose catabolic enzymes were found in bacteria or fungi of GP’s gut. The number of β-glucosidase gene was higher than that of cellulose and cellulose β-1,4-cellobiosidase, and the number of genes from bacteria was more than fungi. Numerous bacteria and fungi may play a key role in assisting the metabolism of cellulose. A large GH genes were found, implying an active metabolism of carbohydrates in the gut of GPs. Among the GH families found, GH13, GH23, and GH3 were obviously abundant. GH13 was the largest of the CAZYs family, wherein many alpha-glucan active enzymes are found and may be closely related with starch hydrolysis (Turkenburg et al., 2009). Starch is one of the most important polysaccharides in bamboo components (Peng et al., 2011). Family GH23 contains lysozymes and soluble lytic transglycosylase (Tufariello et al., 2006). The lytic transglycosylases are an important class of bacterial enzymes as a class of autolysins (Blackburn and Clarke, 2001; Scheurwater et al., 2008). In addition, the lytic transglycosylases may have a role in pathogenesis of some bacterial species (Boneca, 2005). Family GH3 consists primarily of stereochemistry-retaining β-glucosidases and also contains a subfamily of β-*N*-acetylglucosaminidases (Macdonald et al., 2015). Importantly, GH1 and GH2 are gene families with hemicellulose degrading activity, including β-glucosidase, β-galactosidase, β-mannosidase, β-glucuronidase,β-xylosidase, glucan 1,3-β-glucosidase, glucan 1,4-β-glucosidase and xylan 1,4-β-xylosidase, etc. Besides, GH1, GH2, and GH3 are also an important family of enzyme with β-glucosidase activity (Kuntothom et al., 2009; Heins et al., 2011; Talens-Perales et al., 2016) associated with cellulose and hemicellulose degrading activity. Therefore, the study indicated that the microbiota in GPs harbor the ability of cellulose and hemicellulose degradation.

## Conclusion

We provided full details of microbiota structure, including bacteria, fungi, bacteriophages, and helminths based on metagenomic sequencing, in the gut of GPs. Totally, 680 species of bacteria, 198 species of fungi, 185 bacteriophages, and 45 helminths were found. 44 helminths were first reported in the gut of GPs, among which 30 PH were found in mammals or humans suggesting that GPs are important reservoir for parasitic helminths. 1,739 genes encoding cellulase, β-glucosidase, and cellulose β-1,4-cellobiosidase were responsible for the metabolism of cellulose, and 128,707 putative GH genes were found in numerous bacteria and fungi, revealing that the microbiota in GPs harbor the ability of cellulose, hemicellulose and starch hydrolysis. The abundance of *Bacteroidetes* and *Actinobacteria* in wild GPs was significantly higher than that in captive and semiwild GPs (*p* < 0.05). Only a few of bacteria, fungi, bacteriophages, and helminths at genus or species level were found significantly different between the three groups (*p* < 0.05). Taken together, the results indicated not only bacteria but also fungi, bacteriophages, and helminths were diverse in gut of giant pandas providing basis for the further identification of role of gut microbiota. Metagenomics revealed that the bacteria/fungi in gut of GPs harbor the ability of cellulose and hemicellulose degradation. Besides species within the *Clostridium* genus, majority of bacteria and fungi were also found to aid in the digestion of cellulose and hemicellulose in the gut of giant pandas.

## Data Availability

The raw data supporting the conclusions of this manuscript will be made available by the authors, without undue reservation, to any qualified researcher.

## Author Contributions

YH, HZ, and LZ conceived the idea. SY, XG, JM, AZ, YZ, ML, BL, and WD performed the experiments. SY, XG, JM, AZ, YZ, ML, BL, WD, LJ, and SZ performed the statistical analyses. DW, YGH, and CL collected the samples. SY, XG, JM, and AZ wrote the first draft of the manuscript. YH, HZ, SL, and LZ contributed substantially to revisions. All authors read and approved the final manuscript.

## Conflict of Interest Statement

The authors declare that the research was conducted in the absence of any commercial or financial relationships that could be construed as a potential conflict of interest.

## Footnotes

1 http://parasite.wormbase.org/ftp.html

2 http://www.sanger.ac.uk/science/collaboration/50hgp
