# Supplementary material for: Metagenomic Analysis of Bacteria, Fungi, Bacteriophages, and Helminths in the Gut of Giant Pandas
Source: Front Microbiol. 2018 Jul 31;9:1717. doi: 10.3389/fmicb.2018.01717 (PMC6080571; doi:10.3389/fmicb.2018.01717)
Supplement: Supplementary file 1 [file Table_1.DOCX]

**Table S1** **Summary of high-quality reads in each sample**

| Sample ID | High-quality reads (%) | High-quality data (Gb) | High-quality data (%) |
| --- | --- | --- | --- |
| C1 | 96.1 | 10.2 | 95.2 |
| C2 | 94.1 | 10.2 | 95.1 |
| C3 | 96.7 | 10.6 | 96.0 |
| C4 | 97.1 | 10.2 | 96.1 |
| S1 | 99.2 | 9.8 | 98.0 |
| S2 | 99.4 | 8.6 | 97.9 |
| S3 | 97.5 | 9.1 | 95.7 |
| S4 | 96.4 | 4.6 | 99.3 |
| S5 | 94.7 | 9.0 | 94.1 |
| S6 | 97.4 | 4.8 | 99.4 |
| W1 | 94.3 | 12.9 | 94.9 |
| W2 | 99.4 | 8.0 | 98.2 |
| W3 | 94.2 | 16.5 | 93.5 |
| Total | — | 124.6 | — |
| Average | 96.7 | 9.6 | 96.4 |
