# Supplementary material for: Metagenomic Analysis of Bacteria, Fungi, Bacteriophages, and Helminths in the Gut of Giant Pandas
Source: Front Microbiol. 2018 Jul 31;9:1717. doi: 10.3389/fmicb.2018.01717 (PMC6080571; doi:10.3389/fmicb.2018.01717)
Supplement: Supplementary file 2 [file Table_2.DOCX]

**Table S2 Summary of de novo sequencing**

| Sample ID | Min sequence length (bp) | Max sequence length (bp) | Total sequence length (bp) | Sequences greater than 1kb | Total contigs number | Contig N50 | GC content |
| --- | --- | --- | --- | --- | --- | --- | --- |
| C1 | 200 | 197,427 | 35,906,302 | 3865 | 68,582 | 572 | 0.5 |
| C2 | 200 | 53,644 | 56,132,288 | 5,869 | 126,248 | 446 | 0.4 |
| C3 | 200 | 101,453 | 92,559,768 | 11,613 | 183,695 | 529 | 0.5 |
| C4 | 200 | 47,622 | 100,177,140 | 13,421 | 215,307 | 482 | 0.4 |
| S1 | 200 | 142,095 | 33,697,606 | 3,270 | 61,963 | 564 | 0.5 |
| S2 | 200 | 538,259 | 49,216,346 | 7,739 | 63,040 | 1520 | 0.4 |
| S3 | 200 | 44,020 | 40,627,008 | 2,864 | 105,875 | 361 | 0.5 |
| S4 | 200 | 47,363 | 28,570,754 | 4,027 | 62,284 | 483 | 0.4 |
| S5 | 200 | 113,590 | 56,617,585 | 6,798 | 134,754 | 421 | 0.5 |
| S6 | 200 | 214,863 | 90,139,586 | 8,514 | 208,841 | 424 | 0.5 |
| W1 | 200 | 61,923 | 196,016,663 | 29,795 | 344,098 | 604 | 0.5 |
| W2 | 200 | 185,743 | 36,401,901 | 5,117 | 60,539 | 764 | 0.5 |
| W3 | 200 | 408,196 | 383,440,128 | 56,193 | 625,237 | 661 | 0.5 |
| Total | — | — | 1,199,503,075 | 159,085 | 2,260,463 | — | — |
| **Average** | 200 | 165,861 | 92,269,467 | 12,237 | 173,882 | 602 | 0.5 |
