# Supplementary material for: Metagenomic Analysis of Bacteria, Fungi, Bacteriophages, and Helminths in the Gut of Giant Pandas
Source: Front Microbiol. 2018 Jul 31;9:1717. doi: 10.3389/fmicb.2018.01717 (PMC6080571; doi:10.3389/fmicb.2018.01717)
Supplement: Supplementary file 3 [file Table_3.DOCX]

**Table S3 Relative abundance of bacteria, fungi, bacteriophage, and helminths in GP’s gut at phylum or family level**

| Phylum/Family | Relative abundance |
| --- | --- |
| k__Bacteria\|p__Proteobacteria | 75.41% |
| k__Bacteria\|p__Firmicutes | 23.94% |
| k__Bacteria\|p__Bacteroidetes | 0.52% |
| k__Bacteria\|p__Actinobacteria | 0.09% |
| k__Bacteria\|p__Cyanobacteria | 0.02% |
| k__Bacteria\|p__Tenericutes | 0.01% |
| k__Bacteria\|p__Bacteria_noname | 1.10E-05 |
| k__Bacteria\|p__Candidatus_Saccharibacteria | 6.78E-06 |
| k__Bacteria\|p__Acidobacteria | 5.07E-06 |
| k__Bacteria\|p__Spirochaetes | 3.23E-06 |
| k__Bacteria\|p__Deinococcus_Thermus | 3.06E-06 |
| k__Bacteria\|p__Verrucomicrobia | 2.72E-06 |
| k__Bacteria\|p__Fusobacteria | 1.88E-06 |
| k__Bacteria\|p__Gemmatimonadetes | 1.02E-06 |
| k__Fungi\|p__Ascomycota | 75.52% |
| k__Fungi\|p__Basidiomycota | 14.71% |
| k__Fungi\|p__Glomeromycota | 5.35% |
| k__Fungi\|p__Mucoromycota | 2.33% |
| k__Fungi\|p__Microsporidia | 1.99% |
| k__Fungi\|p__Chytridiomycota | 0.09% |
| k__Fungi\|p__Zoopagomycota | 0.01% |
| k__Viruses\|f__Myoviridae | 58.86% |
| k__Viruses\|f__Podoviridae | 9.96% |
| k__Viruses\|f__Siphoviridae | 28.84% |
| k__Viruses\|f__Microviridae | 0.07% |
| k__Viruses\|f__Viruses_noname | 1.97% |
| k__Viruses\|f__Caudovirales_noname | 0.17% |
| k__Viruses\|f__Inoviridae | 0.13% |
| k__Metazoa\|p__Nematoda | 94.82% |
| k__Metazoa\|p__Platyhelminthes | 5.18% |
