# Supplementary material for: Metagenomic Analysis of Bacteria, Fungi, Bacteriophages, and Helminths in the Gut of Giant Pandas
Source: Front Microbiol. 2018 Jul 31;9:1717. doi: 10.3389/fmicb.2018.01717 (PMC6080571; doi:10.3389/fmicb.2018.01717)
Supplement: Supplementary file 4 [file Table_4.DOCX]

**Table S4 Top 10 bacteria, fungi, bacteriophage, and helminths in GP’s gut at genus level**

| Genus | Relative abundance |
| --- | --- |
| k_Bacteria\|g__*Escherichia* | 41.1% |
| k_Bacteria\|g__*Streptococcus* | 15.6% |
| k_Bacteria\|g__*Pseudomonas* | 10.7% |
| k_Bacteria\|g__*Yersinia* | 8.9% |
| k_Bacteria\|g__*Lactococcus* | 4.8% |
| k_Bacteria\|g__*Acinetobacter* | 3.6% |
| k_Bacteria\|g__*Leuconostoc* | 2.1% |
| k_Bacteria\|g__*Stenotrophomonas* | 2.0% |
| k_Bacteria\|g__*Hafnia* | 1.7% |
| k_Bacteria\|g__*Shigella* | 1.6% |
| k__Fungi\|g__*Fusarium* | 22.6% |
| k__Fungi\|g__*Brettanomyces* | 9.6% |
| k__Fungi\|g__*Oidiodendron* | 9.1% |
| k__Fungi\|g__*Tolypocladium* | 5.5% |
| k__Fungi\|g__*Rhizophagus* | 5.4% |
| k__Fungi\|g__*Saccharomyces* | 4.6% |
| k__Fungi\|g__*Piloderma* | 3.4% |
| k__Fungi\|g__*Colletotrichum* | 3.2% |
| k__Fungi\|g__*Hydnomerulius* | 3.0% |
| k__Fungi\|g__*Rhodotorula* | 2.5% |
| k__Viruses\|g__*Lambdavirus* | 22.7% |
| k__Viruses\|g__Myoviridae_noname | 22.6% |
| k__Viruses\|g__*P2virus* | 21.9% |
| k__Viruses\|g__*P1virus* | 13.8% |
| k__Viruses\|g__Siphoviridae_noname | 5.5% |
| k__Viruses\|g__Podoviridae_noname | 3.4% |
| k__Viruses\|g__*Epsilon15virus* | 3.3% |
| k__Viruses\|g__Viruses_noname | 2.0% |
| k__Viruses\|g__*P22virus* | 1.9% |
| k__Viruses\|g__*Nona33virus* | 0.7% |
| k__Metazoa\|g__*Caenorhabditis* | 35.2% |
| k__Metazoa\|g__*Trichuris* | 23.7% |
| k__Metazoa\|g__*Pristionchus* | 7.5% |
| k__Metazoa\|g__*Anisakis* | 5.9% |
| k__Metazoa\|g__*Steinernema* | 4.9% |
| k__Metazoa\|g__*Clonorchis* | 4.3% |
| k__Metazoa\|g__*Toxocara* | 3.0% |
| k__Metazoa\|g__*Ascaris* | 2.8% |
| k__Metazoa\|g__*Globodera* | 2.3% |
| k__Metazoa\|g__*Brugia* | 1.6% |
