# Supplementary material for: Metagenomic Analysis of Bacteria, Fungi, Bacteriophages, and Helminths in the Gut of Giant Pandas
Source: Front Microbiol. 2018 Jul 31;9:1717. doi: 10.3389/fmicb.2018.01717 (PMC6080571; doi:10.3389/fmicb.2018.01717)
Supplement: Supplementary file 5 [file Table_5.DOCX]

**Table S5** **Top 10 bacteria, fungi, bacteriophages, and helminths in GP’s gut at species level**

| Species | Relative abundance |
| --- | --- |
| k__Bacteria\|s__*Escherichia_coli* | 40.8% |
| k__Bacteria\|s__*Yersinia_enterocolitica* | 8.3% |
| k__Bacteria\|s__*Pseudomonas_fluorescens* | 4.9% |
| k__Bacteria\|s__*Lactococcus_lactis* | 4.2% |
| k__Bacteria\|s__*Streptococcus_thermophilus* | 3.5% |
| k__Bacteria\|s__*Streptococcus_infantarius* | 2.9% |
| k__Bacteria\|s__*Streptococcus_gallolyticus* | 2.8% |
| k__Bacteria\|s__*Streptococcus_lutetiensis* | 1.8% |
| k__Bacteria\|s__*Stenotrophomonas_maltophilia* | 1.7% |
| k__Bacteria\|s__*Hafnia_alvei* | 1.7% |
| k__Fungi\|s__*Fusarium_oxysporum* | 17.5% |
| k__Fungi\|s__*Fusarium_proliferatum* | 11.2% |
| k__Fungi\|s__*Brettanomyces_custersianus* | 11.0% |
| k__Fungi\|s__*Oidiodendron_maius* | 9.3% |
| k__Fungi\|s__*Rhizophagus_irregularis* | 6.6% |
| k__Fungi\|s__*Tolypocladium_ophioglossoides* | 6.0% |
| k__Fungi\|s__*Piloderma_croceum* | 3.5% |
| k__Fungi\|s__*Hydnomerulius_pinastri* | 3.4% |
| k__Fungi\|s__*Rhodotorula_toruloides* | 3.3% |
| k__Fungi\|s__*Saccharomyces_pastorianus* | 3.2% |
| k__Viruses\|s__*Escherichia_phage_pro147* | 10.1% |
| k__Viruses\|s__*Shigella_phage_SfIV* | 9.9% |
| k__Viruses\|s__*Enterobacteria_phage_P1* | 8.9% |
| k__Viruses\|s__*Enterobacteria_phage_mEp460* | 6.2% |
| k__Viruses\|s__*Enterobacteria_phage_cdtI* | 5.8% |
| k__Viruses\|s__*Salmonella_phage_SJ46* | 5.0% |
| k__Viruses\|s__*Enterobacteria_phage_SfV* | 3.9% |
| k__Viruses\|s__*Shigella_phage_SfII* | 3.8% |
| k__Viruses\|s__*Enterobacteria_phage_HK629* | 3.5% |
| k__Viruses\|s__*Enterobacteria_phage_SfI* | 3.2% |
| k__Metazoa\|s__*Caenorhabditis_angaria* | 34.0% |
| k__Metazoa\|s__*Trichuris_trichiura* | 23.7% |
| k__Metazoa\|s__*Pristionchus_pacificus* | 7.5% |
| k__Metazoa\|s__*Anisakis_simplex* | 5.9% |
| k__Metazoa\|s__*Clonorchis_sinensis* | 4.3% |
| k__Metazoa\|s__*Steinernema_feltiae* | 3.4% |
| k__Metazoa\|s__*Toxocara_canis* | 3.0% |
| k__Metazoa\|s__*Ascaris_suum* | 2.8% |
| k__Metazoa\|s__*Globodera_pallida* | 2.3% |
| k__Metazoa\|s__*Brugia_timori* | 1.6% |
