# Supplementary material for: Metagenomic Analysis of Bacteria, Fungi, Bacteriophages, and Helminths in the Gut of Giant Pandas
Source: Front Microbiol. 2018 Jul 31;9:1717. doi: 10.3389/fmicb.2018.01717 (PMC6080571; doi:10.3389/fmicb.2018.01717)
Supplement: Supplementary file 6 [file Table_6.DOCX]

**Table S6 GH family more than 1% in GPs**

| GH family | Numbers of gene | Percentage |
| --- | --- | --- |
| GH13 | 12475 | 9.7% |
| GH23 | 12355 | 9.6% |
| GH3 | 10253 | 8.0% |
| GH1 | 6655 | 5.2% |
| GH2 | 6548 | 5.1% |
| GH43 | 4274 | 3.3% |
| GH31 | 3873 | 3.0% |
| GH4 | 3334 | 2.6% |
| GH73 | 3205 | 2.5% |
| GH18 | 3049 | 2.4% |
| GH94 | 2976 | 2.3% |
| GH92 | 2692 | 2.1% |
| GH103 | 2627 | 2.0% |
| GH77 | 2420 | 1.9% |
| GH20 | 2123 | 1.6% |
| GH28 | 2035 | 1.6% |
| GH65 | 2035 | 1.6% |
| GH16 | 2023 | 1.6% |
| GH32 | 1935 | 1.5% |
| GH24 | 1895 | 1.5% |
| GH78 | 1884 | 1.5% |
| GH25 | 1814 | 1.4% |
| GH19 | 1754 | 1.4% |
| GH36 | 1686 | 1.3% |
| GH17 | 1569 | 1.2% |
| GH5 | 1449 | 1.1% |
| GH102 | 1406 | 1.1% |
| Total | 100344 | 78.0% |
