# Supplementary material for: Metagenomic Analysis of Bacteria, Fungi, Bacteriophages, and Helminths in the Gut of Giant Pandas
Source: Front Microbiol. 2018 Jul 31;9:1717. doi: 10.3389/fmicb.2018.01717 (PMC6080571; doi:10.3389/fmicb.2018.01717)
Supplement: Supplementary file 7 [file Table_7.DOCX]

**Table S7 GH family encoding cellulose catabolic enzymes in GPs**

| GH family | Enzyme | Number of genes | Percentage |
| --- | --- | --- | --- |
| GH1 | β-glucosidase | 6655 | 5.17% |
| GH2 | β-glucosidase | 6548 | 5.09% |
| GH3 | β-glucosidase | 10253 | 7.97% |
| GH5 | cellulase, β-glucosidase, cellulose 1,4-β-cellobiosidase | 1449 | 1.13% |
| GH6 | cellulase, cellulose 1,4-β-cellobiosidase | 109 | 0.08% |
| GH7 | cellulase | 69 | 0.05% |
| GH8 | cellulase | 1028 | 0.80% |
| GH9 | cellulase, cellulose 1,4-β-cellobiosidase,β-glucosidase | 583 | 0.45% |
| GH10 | cellulase | 689 | 0.54% |
| GH12 | cellulase | 116 | 0.09% |
| GH26 | cellulase | 359 | 0.28% |
| GH30 | β-glucosidase | 638 | 0.50% |
| GH39 | β-glucosidase | 407 | 0.32% |
| GH44 | cellulase | 17 | 0.01% |
| GH45 | cellulase | 26 | 0.02% |
| GH48 | cellulase | 69 | 0.05% |
| GH51 | cellulase | 1014 | 0.79% |
| GH74 | cellulase | 110 | 0.09% |
| GH116 | β-glucosidase | 70 | 0.05% |
| Total |  | 30209 | 23.47% |
