# Supplementary figures and images for: Metagenomic Analysis of Bacteria, Fungi, Bacteriophages, and Helminths in the Gut of Giant Pandas
Source: Front Microbiol. 2018 Jul 31;9:1717. doi: 10.3389/fmicb.2018.01717 (PMC6080571; doi:10.3389/fmicb.2018.01717)

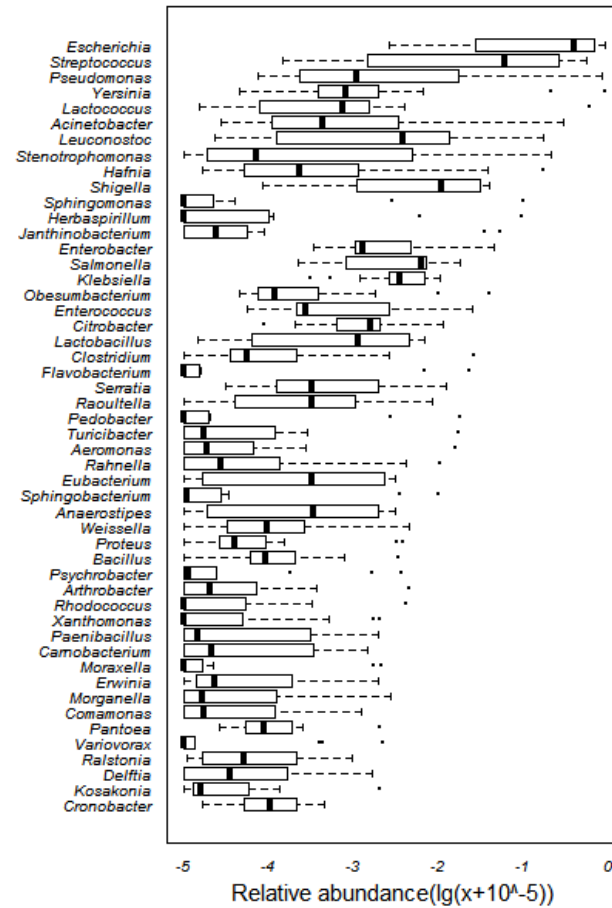

Figure S1 Relative abundance of the 50 most abundant bacterial genera

Supplement: Supplementary file 8 [file Image_1.PDF]

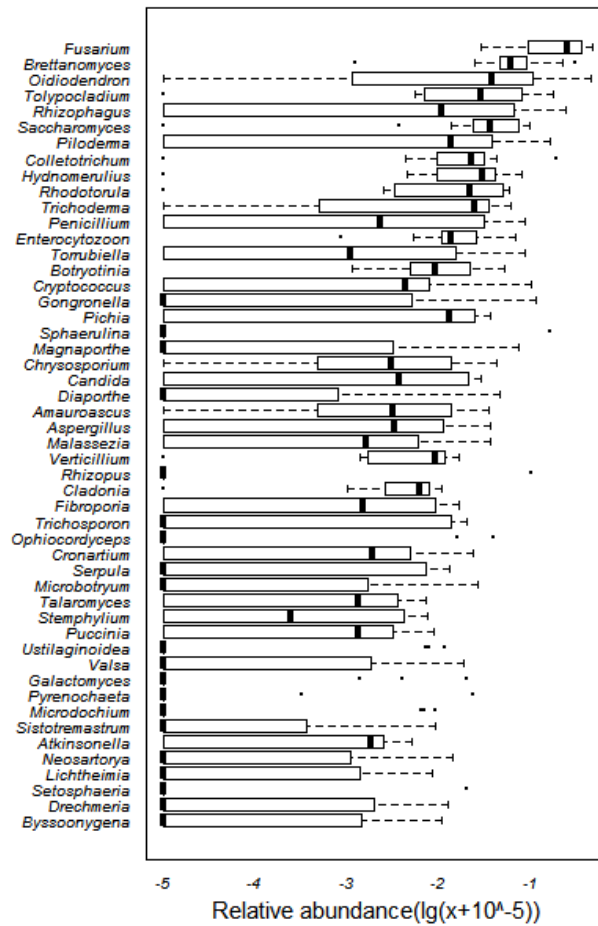

Figure S5 Abundance of the 50 most abundant fungal genera

Supplement: Supplementary file 12 [file Image_5.PDF]

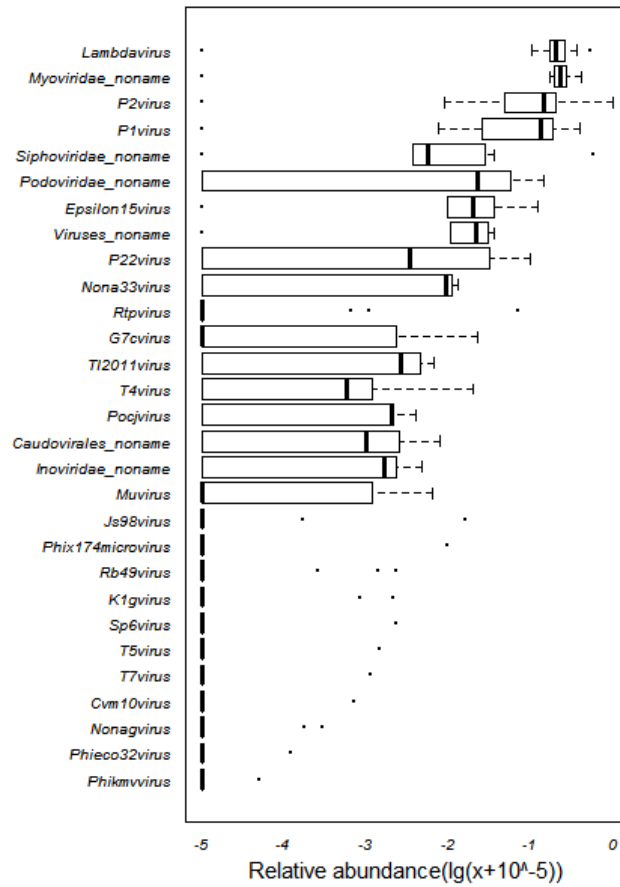

Figure S10 Relative abundance of all 29 phage genera

Supplement: Supplementary file 17 [file Image_10.PDF]

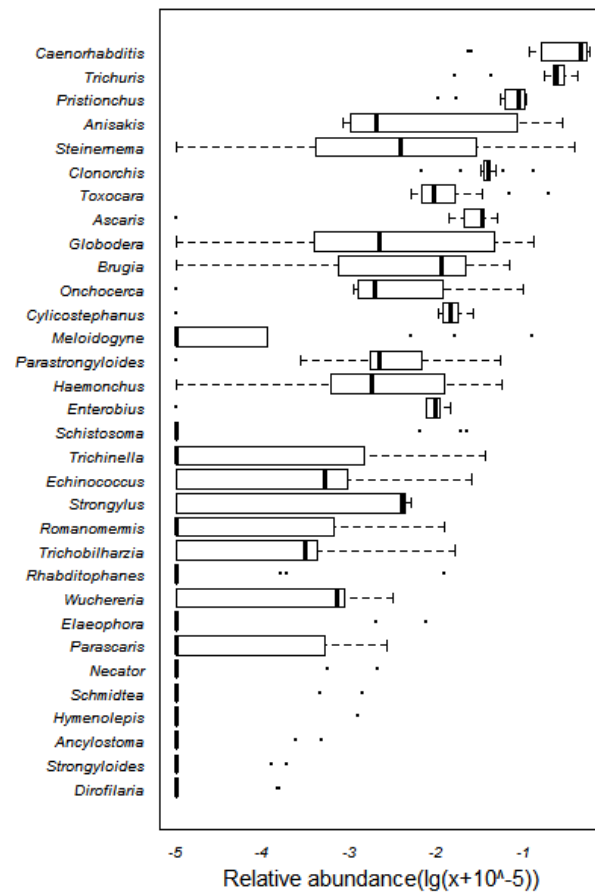

Figure S14 Relative abundance of all helminth genera

Supplement: Supplementary file 21 [file Image_14.PDF]

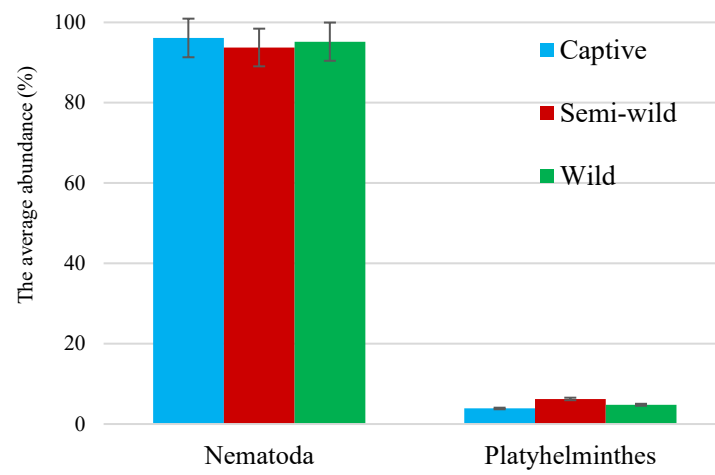

Figure S15 Average abundance of helminths between the three groups at phylum level

Supplement: Supplementary file 22 [file Image_15.pdf]
