# Supplementary material for: Metagenomic Analysis of Bacteria, Fungi, Bacteriophages, and Helminths in the Gut of Giant Pandas
Source: Front Microbiol. 2018 Jul 31;9:1717. doi: 10.3389/fmicb.2018.01717 (PMC6080571; doi:10.3389/fmicb.2018.01717)
Supplement: Supplementary file 10 [file Image_3.PDF]

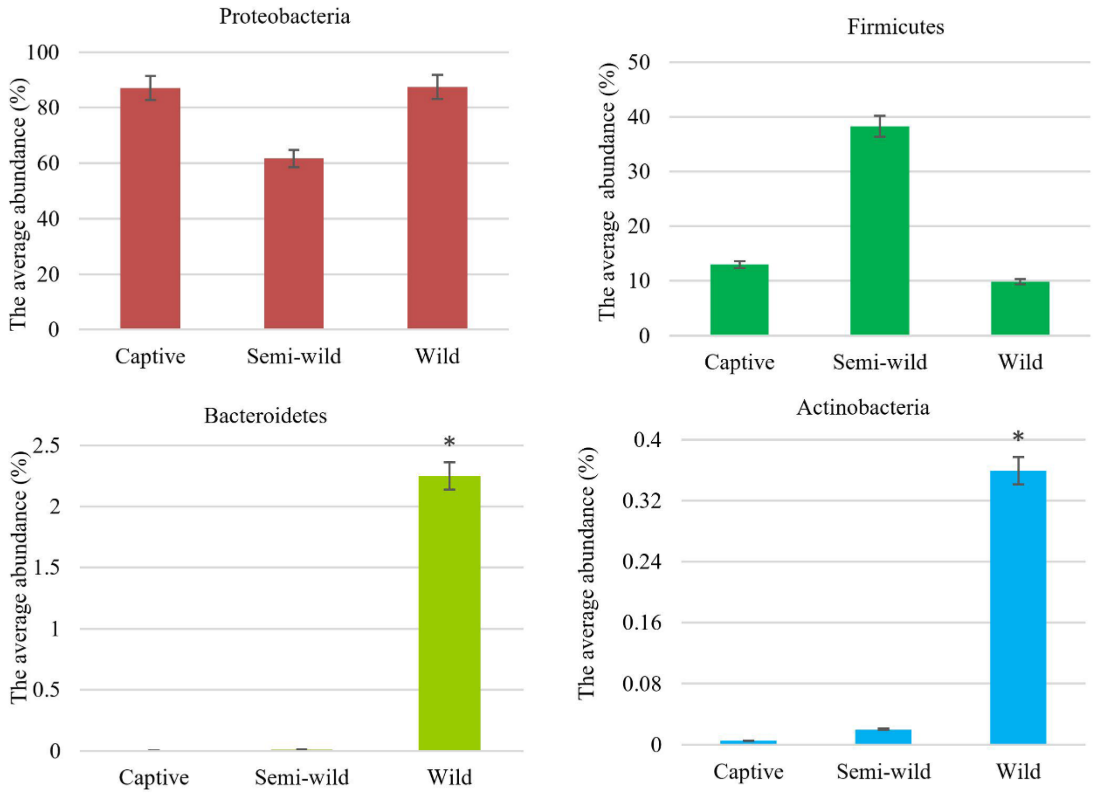

Figure S3 Average abundance of bacteria at phylum level between three different groups of GPs

*, significant difference ($p<0.05$). Cyanobacteria were only detected in semiwild GPs.
