# Supplementary material for: Metagenomic Analysis of Bacteria, Fungi, Bacteriophages, and Helminths in the Gut of Giant Pandas
Source: Front Microbiol. 2018 Jul 31;9:1717. doi: 10.3389/fmicb.2018.01717 (PMC6080571; doi:10.3389/fmicb.2018.01717)
Supplement: Supplementary file 13 [file Image_6.PDF]

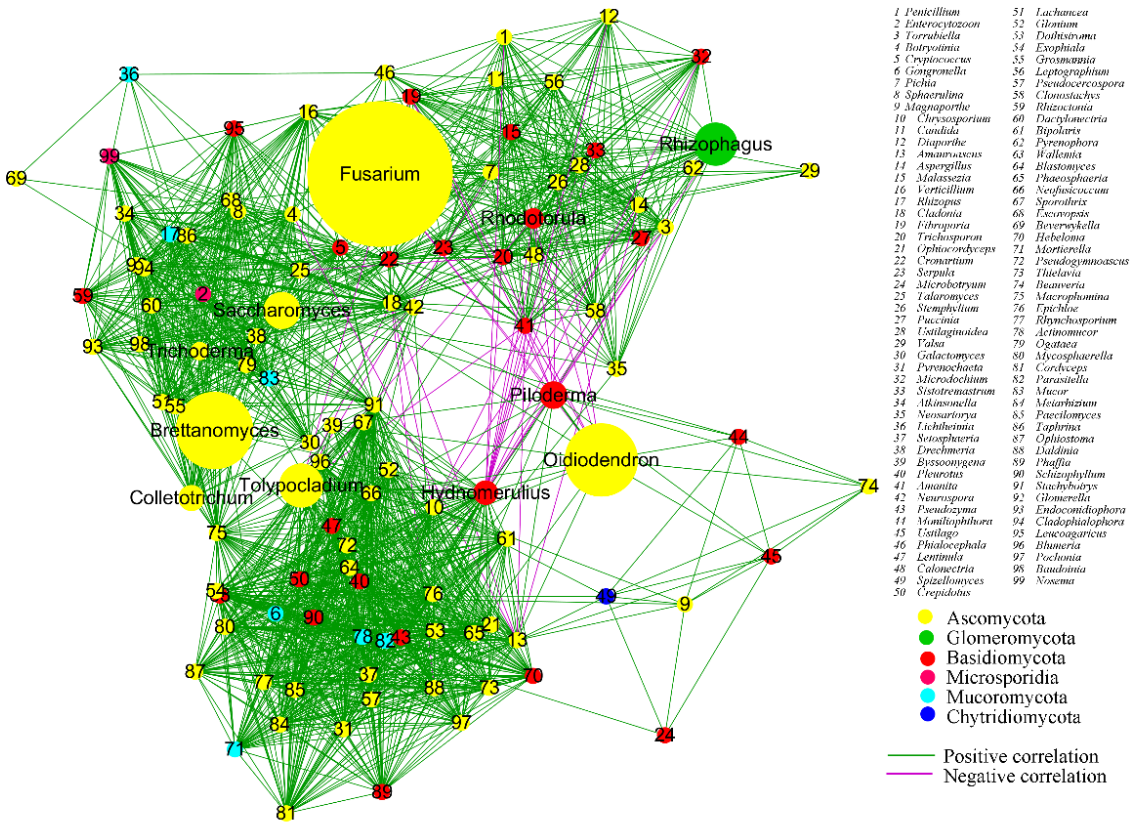

Figure S6 Relations between the most abundant fungal genera

The network was deduced from 1,788 correlations with coefficient of correlation above 0.4 or below −0.4 based on the analysis of 110 fungal genera with an average abundance ≥ 0.01%. Size of the nodes indicates relative abundance of genera. The width of lines indicates the value of coefficient of correlation, and the color of lines indicates the positive or negative correlation.
