# Supplementary material for: Metagenomic Analysis of Bacteria, Fungi, Bacteriophages, and Helminths in the Gut of Giant Pandas
Source: Front Microbiol. 2018 Jul 31;9:1717. doi: 10.3389/fmicb.2018.01717 (PMC6080571; doi:10.3389/fmicb.2018.01717)
Supplement: Supplementary file 14 [file Image_7.PDF]

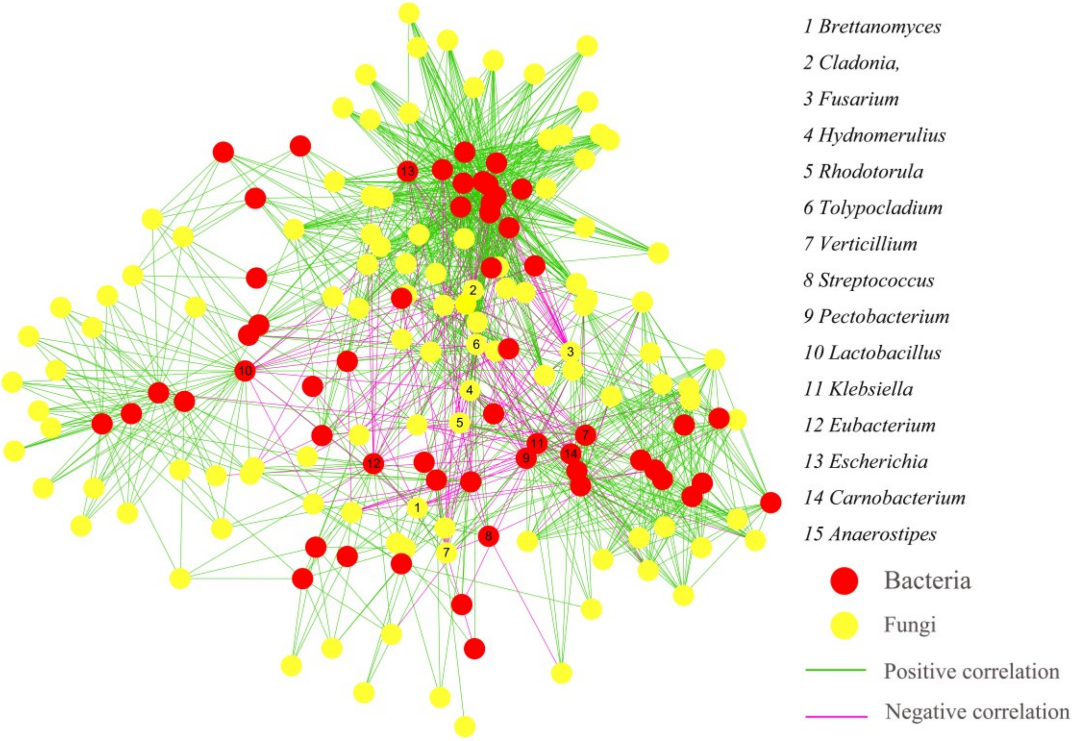

Figure S7 Relations between the most abundant bacterial genera and fungi genera.

The network was deduced from 1231 correlations with coefficient of correlation above 0.4 or below −0.4 based on the analysis of 58 bacterial genera with an average abundance ≥ 0.01% and 110 fungi genera with an average abundance ⩾0.01%. Color of lines indicates the positive or negative correlation.
