# Supplementary material for: Metagenomic Analysis of Bacteria, Fungi, Bacteriophages, and Helminths in the Gut of Giant Pandas
Source: Front Microbiol. 2018 Jul 31;9:1717. doi: 10.3389/fmicb.2018.01717 (PMC6080571; doi:10.3389/fmicb.2018.01717)
Supplement: Supplementary file 18 [file Image_11.PDF]

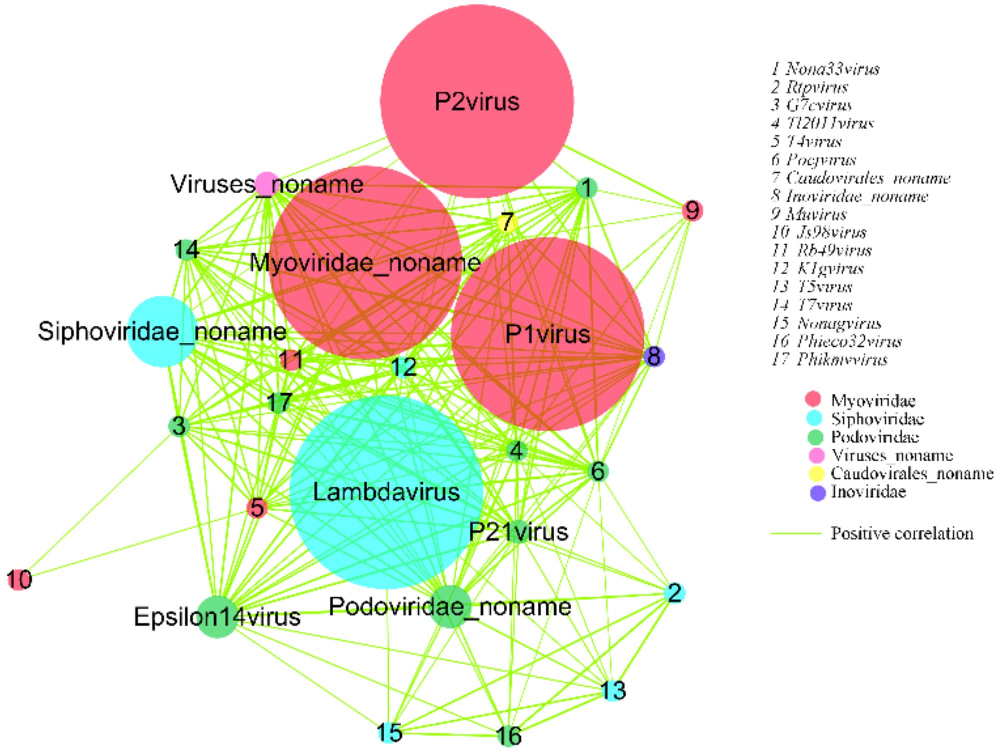


Figure S11 Relations between the most abundant phage genera

The network was deduced from 223 correlations with coefficient of correlation above 0.4 or below −0.4 based on the analysis of 26 phage genera. Size of the nodes indicates genus relative abundance. The width of lines indicates the value of correlation coefficient.
