# Supplementary material for: Metagenomic Analysis of Bacteria, Fungi, Bacteriophages, and Helminths in the Gut of Giant Pandas
Source: Front Microbiol. 2018 Jul 31;9:1717. doi: 10.3389/fmicb.2018.01717 (PMC6080571; doi:10.3389/fmicb.2018.01717)
Supplement: Supplementary file 19 [file Image_12.PDF]

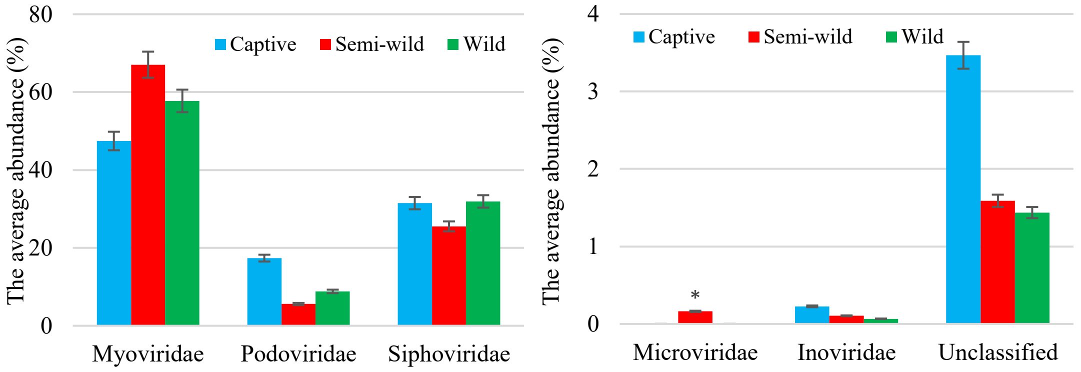

Figure S12 Average abundance of bacteriophage at family level between three different groups of giant pandas

*, significant difference ($p<0.05$).
